# Comparative Analysis of Fecal Microbiota in Healthy Controls and Pancreatic Cancer Patients: A Focus on Tumor Localization Differences in Pancreatic Head and Body–Tail

**DOI:** 10.1002/cam4.71450

**Published:** 2025-12-12

**Authors:** Annacandida Villani, Gandino Mencarelli, Giovanna Cocomazzi, Elena Binda, Edy Virgili, Tiziana Pia Latiano, Evaristo Maiello, Viviana Contu, Francesco Perri, Concetta Panebianco, Valerio Pazienza

**Affiliations:** ^1^ Gastroenterology Unit Fondazione IRCCS “Casa Sollievo Della Sofferenza” San Giovanni Rotondo Italy; ^2^ Cancer Stem Cells Unit, Institute for Stem Cell Biology Regenerative Medicine and Innovative Therapeutics (ISBReMIT), Fondazione IRCCS “Casa Sollievo Della Sofferenza” San Giovanni Rotondo Italy; ^3^ School of Biosciences and Veterinary Medicine, University of Camerino, Camerino, Italy Camerino Italy; ^4^ Oncology Unit Fondazione IRCCS “Casa Sollievo Della Sofferenza Hospital” San Giovanni Rotondo Italy; ^5^ Integrative Medicine Unit Humanitas Gradenigo Torino FC Italy

**Keywords:** dysbiosis, gut microbiota, pancreatic cancer, pancreatic head cancer and pancreatic body‐tail cancer

## Abstract

**Background:**

Pancreatic cancer (PC) remains one of the most lethal malignancies worldwide, characterized by late‐stage diagnosis and a poor prognosis. This study explores the clinical, biochemical, and gut microbiota differences between PC patients and healthy controls (CTRL), as well as between subgroups of PC patients with pancreatic head cancer (PHC) and pancreatic body‐tail cancer (PBTC).

**Methods:**

A total of 72 PC patients and 37 CTRL subjects were included, with further stratification of PC patients into 45 PHC and 27 PBTC cases. Clinical and biochemical data were collected. Gut microbiota was analyzed by 16S rRNA gene sequencing. Alpha‐diversity indices, Firmicutes/Bacteroidetes ratio and taxonomic composition were evaluated and compared in all the experimental group. Correlation analyses were performed between specific bacterial taxa and biochemical markers and a Random Forest algorithm was applied to identify taxa discriminating PC from CTRL and PHC from PBTC.

**Results:**

Clinical and biochemical data revealed significant heterogeneity between groups, with PHC patients exhibiting higher markers of inflammation and liver dysfunction, while PBTC patients showed relatively preserved physiological status. Gut microbiota analysis revealed significant dysbiosis in PC patients compared to CTRL. Alpha‐diversity indices demonstrated reduced species evenness in PC patients, while the Firmicutes/Bacteroidetes ratio was significantly lower. Taxonomic composition analysis indicated enrichment of pro‐inflammatory taxa and depletion of beneficial SCFA‐producing genera. However, subgroup comparisons revealed distinct microbial profiles, with PHC patients enriched in taxa associated with localized inflammation and PBTCs showing higher levels of anti‐inflammatory and SCFA‐producing bacteria. A correlation analysis linked specific bacteria to markers of liver dysfunction and systemic inflammation, such as GGT, ALP, and ESR, while SCFA‐producing taxa correlated negatively with inflammatory markers. A Random Forest algorithm identified key microbial taxa discriminating PC patients from CTRL and PHC from PBTC.

**Conclusions:**

These findings highlight the interplay between microbiota composition, tumor localization, and systemic inflammation, showing a potential for microbiota‐based diagnostics and interventions in PC.

## 1 Introduction

Pancreatic cancer (PC) is one of the most lethal malignancies worldwide, with a critically low 5‐year relative survival rate of only 13% [1]. This poor prognosis is largely attributable to the disease's asymptomatic progression and insidious nature, often leading to diagnosis at advanced stages, when curative options are no longer feasible [2, 3] and palliative systemic chemotherapy remains the standard of care [4, 5, 6]. Several factors hinder early detection, including the lack of specific and reliable biomarkers, the absence of universally accepted screening protocols and the non‐specificity of symptoms in early disease stages [7, 8, 9]. Pancreatic ductal adenocarcinoma (PDAC), the most common subtype of PC, is an exceptionally aggressive malignancy characterized by specific genetic and molecular alterations [10], which are compounded by a highly desmoplastic and immunosuppressive tumor microenvironment (TME), which facilitates immune evasion and therapy resistance [7, 11, 12]. Recent studies have highlighted the critical role of the gut microbiota in modulating cancer biology, including tumor initiation, progression and response to therapy [13, 14]. Specifically, microbial dysbiosis in PC has been linked to chronic inflammation, immune suppression and systemic metabolic dysregulation, all of which are key contributors to tumor development [15, 16, 17]. Furthermore, gut microbiota‐derived metabolites have been shown to influence systemic immunity, angiogenesis and potentially even the behavior of the tumor microenvironment [18]. These findings suggest a dual role for gut microbiota, as both a contributor to tumor progression and a potential therapeutic and diagnostic target in PC [19, 20]. An additional layer of complexity in PC arises from the influence of tumor localization within the pancreas. Tumors located in the pancreatic head, which account for approximately 60%–70% of all cases, exhibit distinct clinical and biological characteristics compared to those located in the body or tail of the pancreas [7, 21]. Pancreatic head tumors are often diagnosed earlier due to their proximity to the bile duct, which can lead to biliary obstruction and jaundice—symptoms that prompt early medical attention [7, 22]. Despite this diagnostic advantage, they are frequently associated with more aggressive local invasion into adjacent critical structures, such as the duodenum, portal vein and superior mesenteric artery. These features complicate surgical resection and contribute to poor outcomes [22]. Conversely, tumors located in the body or tail of the pancreas tend to remain asymptomatic for longer periods, as they are less likely to cause biliary obstruction or other easily recognizable symptoms [11]. Consequently, these tumors are often diagnosed at more advanced stages and are associated with a higher prevalence of distant metastases at the time of detection [23]. Importantly, these anatomical differences are paralleled by distinct biological variations, including differences in vascularization, immune cell infiltration and metabolic interactions within the tumor microenvironment, which may influence tumor progression and overall patient outcomes [11, 24]. Emerging evidence also suggests that the gut microbiota may differentially influence pancreatic tumors depending on their anatomical localization. Tumors in the pancreatic head may interact more directly with the duodenal microbiota, given their anatomical proximity, while those in the body or tail may be more influenced by systemic microbial metabolites and immune‐modulatory factors [24]. Understanding these localization‐specific interactions offers valuable insights into the microbiota's role in PC progression and opens avenues for the development of personalized therapeutic and diagnostic strategies. In this study, we aimed to address two critical gaps in the current understanding of the gut microbiota's role in PC. First, we performed a comparative analysis of the gut microbial composition in PC patients versus control subjects (CTRL) to identify microbial patterns that may serve as diagnostic biomarkers. Second, within the PC cohort, we further stratified patients based on tumor localization: patients with tumors in the pancreatic head (PHC) versus patients with tumors in the body‐tail region (PBTC). This stratification enabled us to explore how tumor localization influences gut microbiota composition and its potential implications for tumor biology, progression and clinical outcome. In addition, we aimed to identify specific microbial signatures associated with PC that reflect both the presence of the tumor and its anatomical localization. These findings could lay the groundwork for the development of novel diagnostic tools and microbiota‐targeted therapies, ultimately improving outcomes for PC patients.

## 2 Results

### 2.1 Sample Characteristics

A total of 72 patients diagnosed with pancreatic tumors were included in this study (mean age at enrollment: 67.8 years), along with 37 healthy controls (mean age at enrollment: 62.3 years). PC patients were further stratified into two subgroups: 45 individuals with PHC (mean age at enrollment: 68.2 years) and 27 individuals with PBTC (mean age at enrollment: 65.7 years). Anthropometric and lifestyle data for PC patients and CTRL subjects are summarized in Table 1, while anthropometric, lifestyle and clinical data specific to PHC and PBTC patients are detailed in Table 2. In the comparison between PC patients and CTRL subjects, the study participants were well‐matched as regards BMI, smoking habits and alcohol consumption. Slight though significant differences concerned the age at enrollment, with PC patients being older than controls, despite both groups of subjects being on average in their 60s, and the gender distribution, with a higher proportion of males in the PC group compared to the CTRL group. Differences in dietary patterns were also observed, with a higher percentage of PC patients following a diabetic/hypoglucidic diet, as expected. When comparing PHC and PBTC patients, significant differences were identified in some tumor‐related characteristics and biochemical parameters. Jaundice and endoprosthesis placement were more prevalent in PHC patients than in PBTC patients, with 57.8% vs. 0% for jaundice and 60% vs. 11.1% for endoprosthesis, respectively. Blood biochemical parameters at enrollment also varied significantly between the groups. Red blood cell count and albumin levels were higher in PBTC patients. On the contrary, PHC patients had higher levels of bilirubin, AST, ALT, GGT, ALP, aPTT, lipase and ESR.

**TABLE 1:** Characteristics of patients with pancreatic cancer (PC) with respect to healthy subjects (CTRL) at the enrollment.

Variable	Category	All subjects (*N* = 109)	PC (*N* = 72)	CTRL (*N* = 37)	*p*
*Clinical characteristics*					
Age at enrollment (years)	Mean ± SD	65.9 ± 9.2	67.8 ± 9.6	62.3 ± 7	0.003^a^
Gender, *N* (%)	Males	60 (55.0)	45 (62.5)	15 (40.5)	0.042^b^
BMI at enrollment (kg/m^2^)	Median [IQR]	25.0 [22.9–28.3]	24.4 [22.7–27.9]	26.3 [23.7–28.7]	0.328^c^
*Lifestyle info*					
Smoking status, *N* (%)	No	67 (61.5)	40 (55.5)	27 (73.0)	0.056^d^
	Former smoker	13 (11.9)	12 (16.7)	1 (2.7)	
	Current smoker	24 (22.0)	18 (25.0)	6 (16.2)	
	N/A	5 (4.6)	2 (2.8)	3 (8.1)	
Diet, *N* (%)	Diabetic/hypoglucidic	14 (12.8)	13 (18.1)	1 (2.7)	0.015^d^
	Free/hyperglucidic/hyperproteic	9 (8.3)	9 (12.5)	0 (0.0)	
	Mediterranean	80 (73.4)	46 (63.9)	34 (91.9)	
	Vegetarian	1 (0.9)	1 (1.4)	0 (0.0)	
	N/A	5 (4.6)	3 (4.2)	2 (5.4)	
Alcohol consumption, *N* (%)	No	93 (85.3)	64 (88.9)	29 (78.4)	0.340^d^
	Yes	10 (9.2)	5 (6.9)	5 (13.5)	
	N/A	6 (5.5)	3 (4.2)	3 (8.1)	

Abbreviations: CTRL, healthy subjects; IQR, interquartile range (i.e., first‐third quartiles); N/A, not available; PC, patients with pancreatic tumor at enrollment; SD, standard deviation.

^a^ *p*‐value from the two‐sample test.

^b^ *p*‐value from chi‐squared test with Yates' continuity correction.

^c^ *p*‐value from Mann–Whitney *U* test.

^d^ *p*‐value from chi‐squared test.

**TABLE 2:** Characteristics of patients with pancreatic head cancer (PHC) and patients with pancreatic body‐tail cancer (PBTC) at the enrollment.

Variable	Category	All subjects (*N* = 72)	PHC (*N* = 45)	PBTC (*N* = 27)	*p*
*Clinical characteristics*					
Age at enrollment (years)	Mean ± SD	67.7 ± 9.6	68.2 ± 9.7	65.7 ± 9.2	0.189^a^
Gender, *N* (%)	Males	45 (62.5)	28 (62.2)	17 (63.0)	> 0.999^b^
BMI at enrollment (kg/m^2^)	Median [IQR]	24.4 [22.7–27.9]	23.9 [22.7–27.1]	25.9 [23.1–28.1]	0.274^c^
Comorbidities, *N* (%)	Yes	58 (80.6)	37 (82.2)	21 (77.8)	0.878^b^
	Cardiovascular disease	49/58 (84.5)	30/37 (81.1)	19/21 (90.5)	0.567^b^
	Diabetes	30/58 (51.7)	20/37 (54.0)	10/21 (47.6)	0.843^b^
	Hypercholesterolemia/Dyslipidemia	6/58 (10.3)	4/37 (10.8)	2/21 (9.5)	> 0.999^d^
	Others	28/58 (48.3)	20/37 (54.0)	8/21 (38.1)	0.371^b^
*Tumor characteristics, N (%)*					
Tumor stage	IB	2 (2.8)	2 (4.4)	0 (0.0)	0.110^e^
	IIA/IIB	2 (2.8)	1 (2.2)	1 (3.7)	
	III	1 (1.4)	0 (0.0)	1 (3.7)	
	IV	37 (51.4)	19 (42.2)	18 (66.7)	
	N/A	30 (41.7)	23 (51.1)	7 (25.9)	
Metastases	Yes	43 (59.7)	23 (51.1)	20 (74.1)	0.094^b^
Other tumors	No	53 (73.6)	33 (73.3)	20 (74.1)	0.211^e^
	Yes	17 (23.6)	10 (22.2)	7 (25.9)	
	N/A	2 (2.8)	0 (0.0)	2 (7.4)	
Family history of cancer	Yes	24 (33.3)	15 (33.3)	9 (33.3)	> 0.999^d^
Jaundice	Yes	26 (36.1)	26 (57.8)	0 (0.0)	< 0.0001^d^
Endoprosthesis	Yes	30 (41.7)	27 (60.0)	3 (11.1)	< 0.0001^d^
*Concomitant drugs (taken during the enrollment visit), N (%)*					
Antihypertensive anticoagulant cardioprotective drugs	Yes	34 (47.2)	21 (46.7)	13 (48.1)	> 0.999^d^
Antidiabetic drugs pancreatic enzymes	Yes	21 (29.2)	15 (33.3)	6 (22.2)	0.424^d^
Statins	Yes	7 (9.7)	3 (6.7)	4 (14.8)	0.413^d^
Other drugs	Yes	21 (29.2)	14 (31.1)	7 (25.9)	0.790^d^
*Lifestyle info, N (%)*					
Smoking status	No	40 (55.6)	26 (57.8)	14 (51.8)	0.873^e^
	Former smoker	12 (16.7)	8 (17.8)	4 (14.8)	
	Current smoker	18 (25)	10 (22.2)	8 (29.6)	
	N/A	2 (2.8)	1 (2.2)	1 (3.7)	
Diet	Diabetic/hypoglucidic	14 (19.4)	7 (15.6)	7 (25.9)	0.313^e^
	Free/hyperglucidic/hyperproteic	8 (11.1)	7 (15.6)	1 (3.7)	
	Mediterranean	46 (63.9)	29 (64.4)	17 (63.0)	
	Vegetarian	1 (1.4)	1 (2.2)	0 (0.0)	
	N/A	3 (4.2)	1 (2.2)	2 (7.4)	
Alcohol consumption	No	64 (88.9)	39 (86.7)	25 (92.6)	0.690^e^
	Yes	5 (6.9)	4 (8.9)	1 (3.7)	
	N/A	3 (4.2)	2 (4.4)	1 (3.7)	
*Biochemical exams at enrollment visit*					
Hemoglobin (g/dL)	Median [IQR]	12.7 [11.2–14.0]	12.6 [10.2–13.7]	13.1 [11.9–14.1]	0.063^c^
RBC (M/μL)	Median [IQR]	4.4 [4.0–4.7]	4.3 [3.5–4.7]	4.5 [4.2–4.7]	0.052^c^
PLT (K/μL)	Median [IQR]	234.0 [185.2–298.5]	246.0 [188.0–298.0]	229.0 [177.5–292.5]	0.679^c^
WBC (K/μL)	Median [IQR]	7.2 [5.6–9.2]	7.0 [5.4–9.1]	7.7 [5.9–9.3]	0.350^c^
Neutrophils (K/μL)	Median [IQR]	4.8 [3.4–6.2]	4.4 [3.4–6.0]	5.1 [3.5–6.4]	0.447^c^
Eosinophils (K/μL)	Median [IQR]	0.1 [0.1–0.2]	0.1 [0.1–0.2]	0.1 [0.1–0.2]	0.667^c^
Basophils (K/μL)	Median [IQR]	0.0 [0.0–0.0]	0.0 [0.0–0.1]	0.0 [0.0–0.0]	0.506^c^
Lymphocytes (K/μL)	Median [IQR]	1.5 [1.1–2.0]	1.5 [1.0–2.0]	1.5 [1.1–2.0]	0.504^c^
Monocytes (K/μL)	Median [IQR]	0.4 [0.4–0.6]	0.4 [0.3–0.6]	0.4 [0.4–0.6]	0.658^c^
Glycemia (mg/dL)	Median [IQR]	109.5 [90.0–162.7]	114.0 [89.0–182.5]	107.0 [93.0–124.0]	0.306^c^
Total Protein (g/dL)	Median [IQR]	6.6 [6.3–7.2]	6.6 [6.1–7.0]	6.8 [6.4–7.3]	0.107^c^
Albumin (g/dL)	Median [IQR]	3.4 [2.8–3.8]	3.2 [2.7–3.7]	3.7 [3.4–4.2]	0.002^c^
Bilirubin (mg/dL)	Median [IQR]	0.8 [0.5–7.6]	4.2 [0.8–10.0]	0.5 [0.4–0.6]	< 0.000001^c^
AST (IU/L)	Median [IQR]	34.0 [17.0–88.7]	71.0 [33.0–128.0]	17 [13.0–24.5]	< 0.000001^c^
ALT (IU/L)	Median [IQR]	40.0 [21.0–154.0]	122.0 [40.0–200.0]	21.0 [17.5–30.0]	< 0.000001^c^
GGT (IU/L)	Median [IQR]	212.0 [27.0–555.5]	425.0 [206.0–919.0]	25.0 [18.0–95.0]	< 0.000001^c^
ALP (IU/L)	Median [IQR]	184.0 [73.5–384.7]	348.0 [198.0–489.0]	73.0 [60.5–107.0]	< 0.000001^c^
aPTT (SEC)	Median [IQR]	24.5 [23.1–25.7]	24.9 [24.0–26.0]	23.7 [22.3–25.0]	0.035^c^
PT (INR)	Median [IQR]	1.1 [1.0–1.1]	1.1 [1.0–1.1]	1.1 [1.0–1.1]	0.123^c^
Pancreatic amylase (IU/L)	Median [IQR]	28.0 [17.0–39.2]	31.0 [16.0–63.0]	21.0 [18.0–23.0]	0.092^c^
Lipase (U/L)	Median [IQR]	157.0 [75.0–419.0]	223.5 [99.0–612.2]	110.0 [52.0–140.0]	0.005^c^
Creatinine (mg/dL)	Median [IQR]	0.7 [0.6–0.9]	0.7 [0.6–0.9]	0.7 [0.7–1.0]	0.573^c^
CEA (ng/mL)	Median [IQR]	3.1 [1.3–9.4]	2.4 [1.3–8.1]	3.9 [1.5–13.6]	0.243^c^
CA 19–9 (U/mL)	Median [IQR]	312.6 [43.8–2912.1]	319.7 [51.7–2912.1]	160.2 [14.8–3154.45]	0.594^c^
ESR (mm)	Median [IQR]	47.0 [20.0–71.5]	64.0 [35.0–78.0]	18.0 [14.7–28.5]	0.0002^c^
CRP (mg/dL)	Median [IQR]	1.7 [0.7–2.9]	1.5 [0.7–2.7]	2.3 [0.8–3.9]	0.682^c^

Abbreviations: CT, chemotherapy; IQR, interquartile range (i.e., first‐third quartiles); N/A, not available; PBTC, patients with pancreatic body‐tail tumor at enrollment; PHC, patients with pancreatic head tumor at enrollment; RT, radiotherapy; SD, standard deviation.

^a^ *p*‐value from the two‐sample test.

^b^ *p*‐value from chi‐squared test with Yates' continuity correction.

^c^ *p*‐value from Mann–Whitney *U* test.

^d^ *p*‐value from Fisher's exact test.

^e^ *p*‐value from chi‐square test.

### 2.2 Comparison of Fecal Microbiota Composition Between PC Patients and CTRL Subjects

**FIGURE 1:**
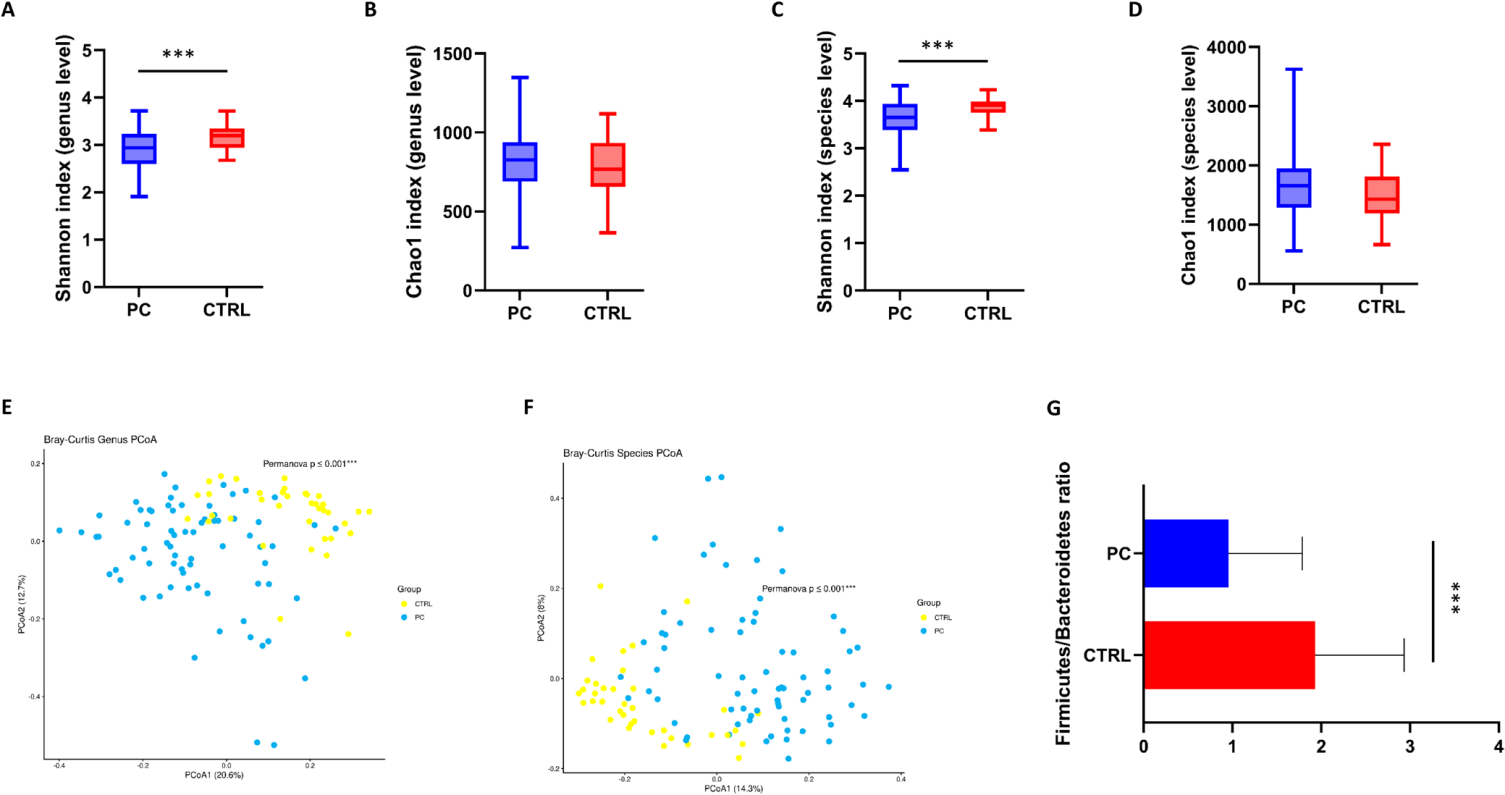
Boxplots of Shannon index (evenness) and Chao1 index (richness) calculated at the genus level (A and B respectively) and at the species level (C and D respectively) in all PC patients versus CTRL group. PCoA plots illustrating beta‐diversity derived from Bray–Curtis distances comparing the sample distribution between PC and CTRL group, calculated at the genus and species level, respectively (E and F respectively). Panel G shows barplots of the Firmicutes/Bacteroidetes ratio in PC patients with respect to control group. ***p < 0.001.

For all the 109 study participants, 16S rRNA gene sequencing yielded an average of 244,032.24 (SD ± 201,901.12) read pairs. To examine the within‐sample diversity of the bacterial profiles, alpha‐diversity indices (Shannon and Chao1) were calculated at the genus and species levels. When comparing PC patients to CTRL subjects, a statistically significant reduction in evenness in the former with respect to the latter, was observed at both taxonomic levels, as measured by the Shannon index (*p* = 0.0006 at the genus level and *p* = 0.0004 at the species level) (Figure 1). As for beta‐diversity, instead, PERMANOVA tests based on Bray–Curtis distances revealed significant differences between groups at both the genus (Figure 1E) and species (Figure 1F) levels (*p* ≤ 0.001).

**FIGURE 2:**
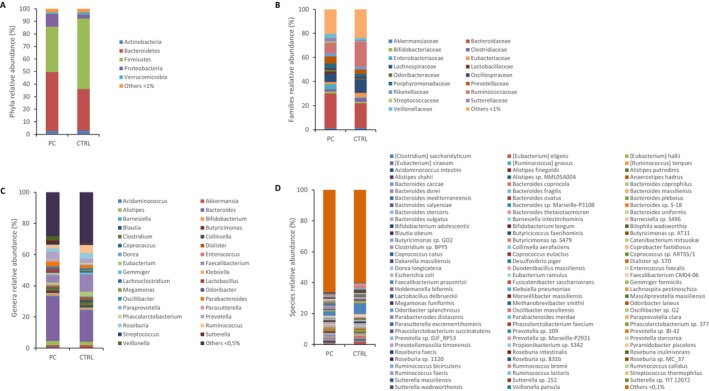
Gut microbiota composition at different taxonomic levels grouped by pancreatic cancer (PC) patients and healthy subjects (CTRL). Mean relative abundance (%) at phylum (A), family (B), genus (C) and species (D) levels in PC patients and the CTRL group, respectively. The “Others” category includes all bacteria whose mean relative abundance is less than 1% at the phylum and family levels and less than 0.5% at the genus level and less than 0.1% at the species level.

Next, the fecal microbiota profile of both PC and CTRL was analyzed. As expected, the two most prevalent phyla—Firmicutes and Bacteroidetes—accounted for almost 80% of all bacteria in both groups. The Firmicutes/Bacteroidetes (F/B) ratio showed a mean value of 1.69 in healthy subjects and 0.77 in patients with PC, revealing a statistically significant reduction in the PC group compared to healthy participants (Figure 1G). Figure 2 represents the composition of the fecal microbiota of PC and CTRL subjects at the phylum (A), family (B), genus (C) and species levels (D). Significant differences between the two groups emerged at all taxonomic levels, as detailed in Table S1. At the phylum level, Bacteroidetes (46.97% vs. 33.24%), Fusobacteria (0.22% vs. 0.02%), Proteobacteria (9.94% vs. 3.17%) and Synergistetes (0.47% vs. 0.09%) were enriched, while Euryarchaeota (0.01% vs. 0.14%) and Firmicutes (36.30% vs. 56.08%) were reduced in PC versus CTRL. Among the families differentially represented, a significant increase in the abundance of Bacteroidaceae (28.98% vs. 20.43%), Enterobacteriaceae (4.16% vs. 0.81%), Enterococcaceae (0.61% vs. 0.02%) and Fusobacteriaceae (0.21% vs. 0.02%) was found, while a lower abundance of Lachnospiraceae (6.90% vs. 11.08%) and Ruminococcaceae (8.58% vs. 20.48%) was found in PC patients versus CTRL. At the genus level, *Escherichia* (0.27% vs. 0.09%), *Fusobacterium* (0.19% vs. 0.02%), *Klebsiella* (0.91% vs. 0.02%) and *Lactobacillus* (1.62% vs. 0.08%) were some of the bacteria enriched whereas *Faecalibacterium* (4.53% vs. 11.09%) and *Ruminococcus* (2.23% vs. 5.16%) were among the depleted in patients as compared to healthy subjects. Finally, a rise in *Bacteroides fragilis* (0.75% vs. 0.20%), *Enterococcus faecalis* (0.29% vs. 0.004%), *Escherichia coli* (0.15% vs. 0.49%), *Fusobacterium nucleatum* (0.06% vs. 0.0004%), *Klebsiella pneumoniae* (0.18% vs. 0.006%) and several species belonging to *Lactobacillus* and, conversely, a drop in *Methanobrevibacter smithii* (0.004% vs. 0.11%) and *Roseburia intestinalis* (0.11% vs. 0.53%) were among the significant changes observed when comparing PC to CTRL at the species level.

### 2.3 Random Forest Classification for Fecal Microbiota in PC Patients and CTRL Subjects

**FIGURE 3:**
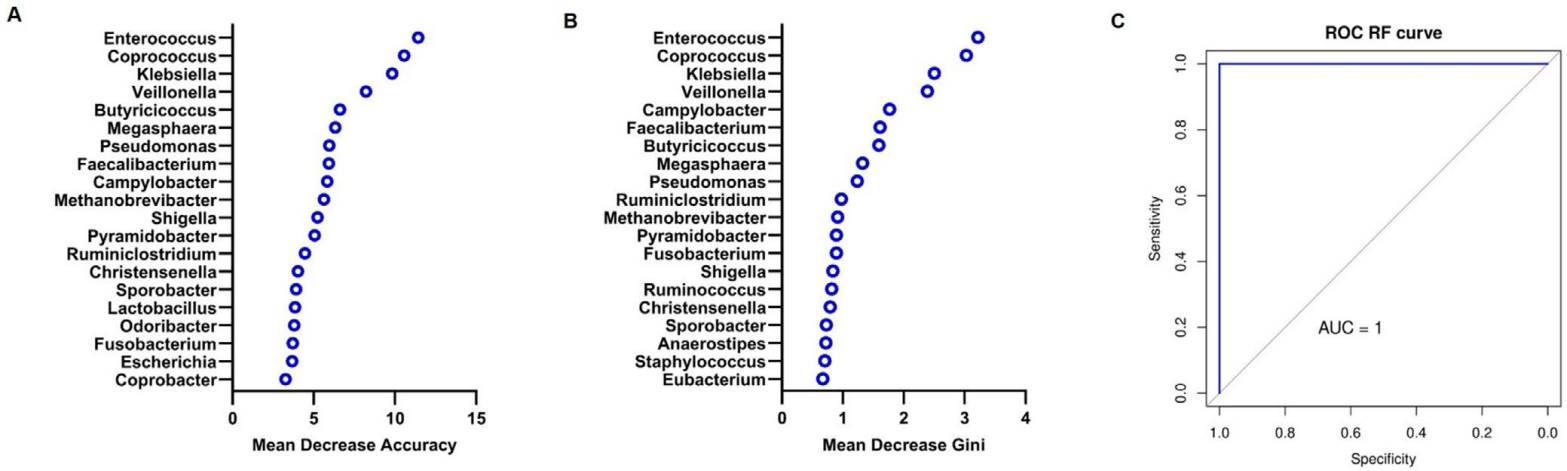
Random Forest model for PC and CTRL classification at the genus level. Mean Decrease Accuracy measure of the top 20 most important genera in predicting the group. The higher the values, the more the taxon is crucial for the prediction accuracy of the model (A). Mean Decrease Gini of the top 20 most important genera. The higher the value, the more the bacterium contributes to creating pure, homogeneous nodes within the decision trees (B). The performance of the model was assessed through an AUC analysis (C).

A Random Forest algorithm was applied to obtain a microbial classifier in PC patients and healthy controls. Figure 3A,B represent the variable importance plot of the top 20 bacterial genera ordered according to the Mean Decrease Accuracy and to the Mean Decrease Gini, respectively. In both cases, *Enterococcus, Coprococcus*, *Klebsiella* and *Veillonella* showed the most influence in classification between PC and CTRL subjects. As depicted in Figure 3C, this model showed a perfect predictive performance, producing an area under the ROC curve (AUC) of 1 (95% CI: 1.000–1.000), with 100% specificity and sensitivity.

### 2.4 Comparison of Fecal Microbiota Composition Between PHC Patients and PBTC Group

**FIGURE 4:**
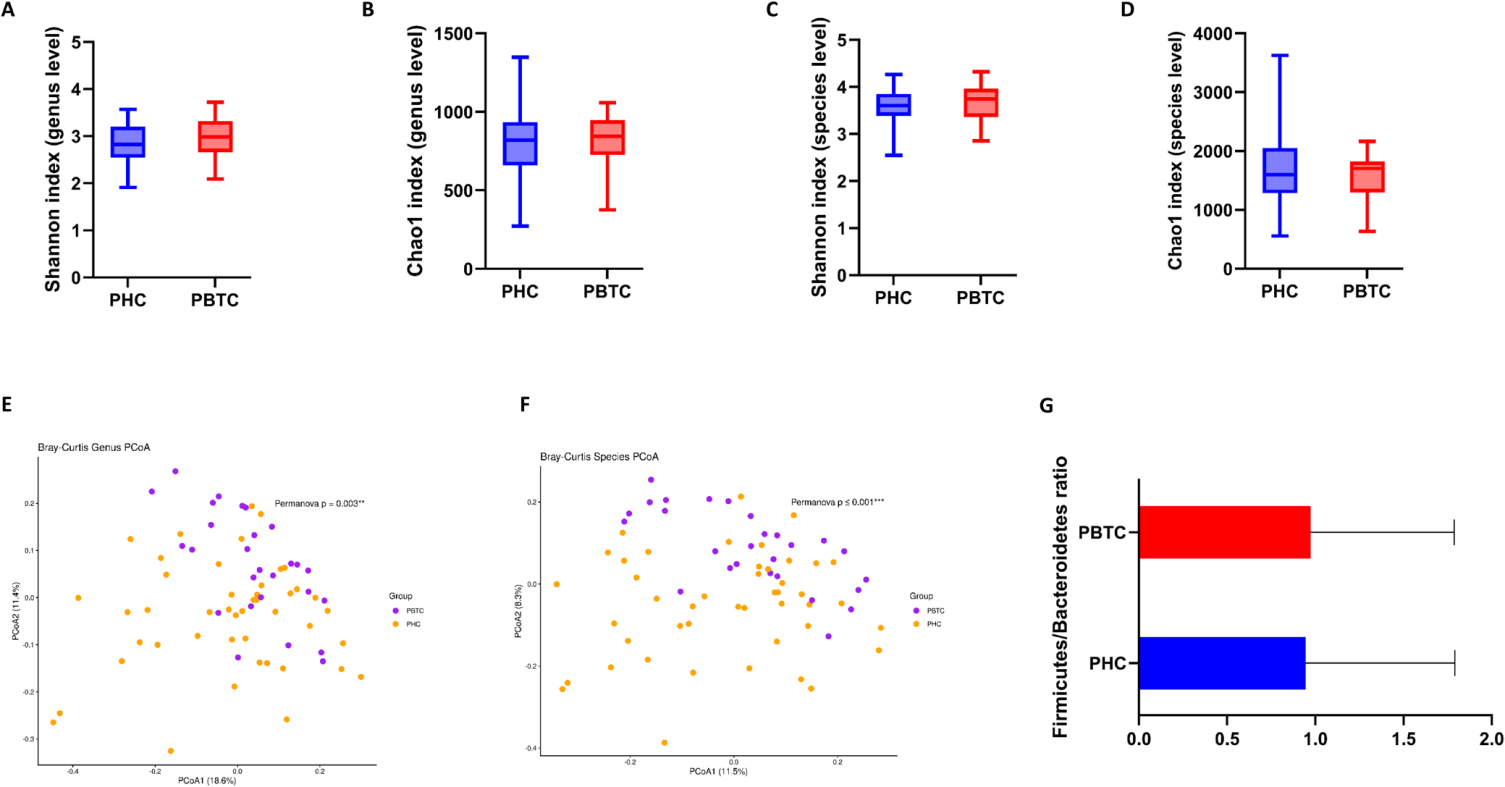
Boxplots of Shannon index (evenness) and Chao1 index (richness) calculated at the genus level (A and B respectively) and at the species level (C and D respectively) in all PHC patients versus PBTC group. PCoA plots illustrating beta‐diversity derived from Bray–Curtis distances comparing the sample distribution between PHC and PBTC group, calculated at the genus and species level, respectively (E and F respectively). Panel G shows barplots of the Firmicutes/Bacteroidetes ratio in PHC patients versus PBTC subjects.

**FIGURE 5:**
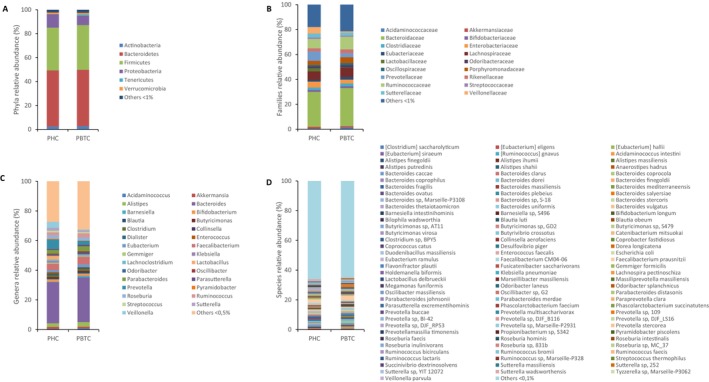
Gut microbiota composition at different taxonomic levels grouped by pancreatic cancer patients with head tumor localization (PHC) and pancreatic cancer patients with body‐tail tumor localization (PBTC). Mean relative abundance (%) at phylum (A), family (B), genus (C) and species (D) levels in PHC and PBTC patients, respectively. The “Others” category includes all bacteria whose mean relative abundance is less than 1% at the phylum and family levels, less than 0.5% at the genus level, and less than 0.5% at the species level.

An average of 25,9131.21 (SD ±22,4605.52) read pairs were obtained from 16S rRNA gene sequencing for all the 72 PC patients. Alpha‐diversity indices (Shannon and Chao1) were calculated at the genus and species levels to assess the within‐sample diversity of the bacterial profiles; however, no statistically significant differences were observed between PHC and PBTC patients (Figure 4A–D). As for beta‐diversity, PERMANOVA tests based on Bray–Curtis distances showed significant differences between the two groups at both the genus (Figure 4E) (*p* = 0.003) and species (Figure 4F) levels (*p* ≤ 0.001). No statistically significant difference was observed in the Firmicutes/Bacteroidetes ratio (Figure 4G). The compositional profiles at the phylum (A), family (B), genus (C) and species levels (D) are displayed in Figure 5. Significant variations were found between the two groups at all taxonomic levels, as fully listed in Table S2. Tenericutes was the only phylum enriched in the PBTC group as compared to the PHC group (0.07% vs. 1.17%). At the family level, Desulfovibrionaceae (0.28% vs. 0.70%) and Erysipelotrichaceae (0.30% vs. 0.51%) were significantly higher while Enterococcaceae (0.91% vs. 0.11%), Fusobacteriaceae (0.31% vs. 0.05%), Lactobacillaceae (2.47% vs. 0.30%), Staphylococcaceae (0.02% vs. 0.003%), Veillonellaceae (5.24% vs. 0.97%) and Yersiniaceae (0.06% vs. 0.02%) were among the bacteria decreased in the PBTC group versus PHC subjects. At the genus level, *Anaeromassilibacillus* (0.005% vs. 0.01%), *Bilophila* (0.13% vs. 0.41%), *Intestinibacter* (0.01% vs. 0.06%) and *Negativibacillus* (0.02% vs. 0.04%), were some of the taxa increased in PBTC patients with respect to PHC patients. On the contrary, *Campylobacter* (0.02% vs. 0.008%), *Catenibacterium* (0.20% vs. 0.03%), *Enterobacter* (0.08% vs. 0.03%), *Enterococcus* (0.88% vs. 0.11%), *Fusobacterium* (0.29% vs. 0.02%), *Klebsiella* (1.32% vs. 0.23%), *Lactobacillus* (2.42% vs. 0.29%), and *Veillonella* (4.01% vs. 0.52%) were among the genera decreased in the PBTC versus PHC patients. Going down in the taxonomic hierarchy, an interesting decrease in *Enterococcus faecalis* (0.44% vs. 0.03%), *Fusobacterium nucleatum* (0.09% vs. 0.0002%), *Lactobacillus gasseri* (0.05% vs. 0.03%), and in different *Prevotella* species was observed when comparing PBTC to PHC patients.

### 2.5 Random Forest Classification for Fecal Microbiota in PHC and PBTC Patients

**FIGURE 6:**
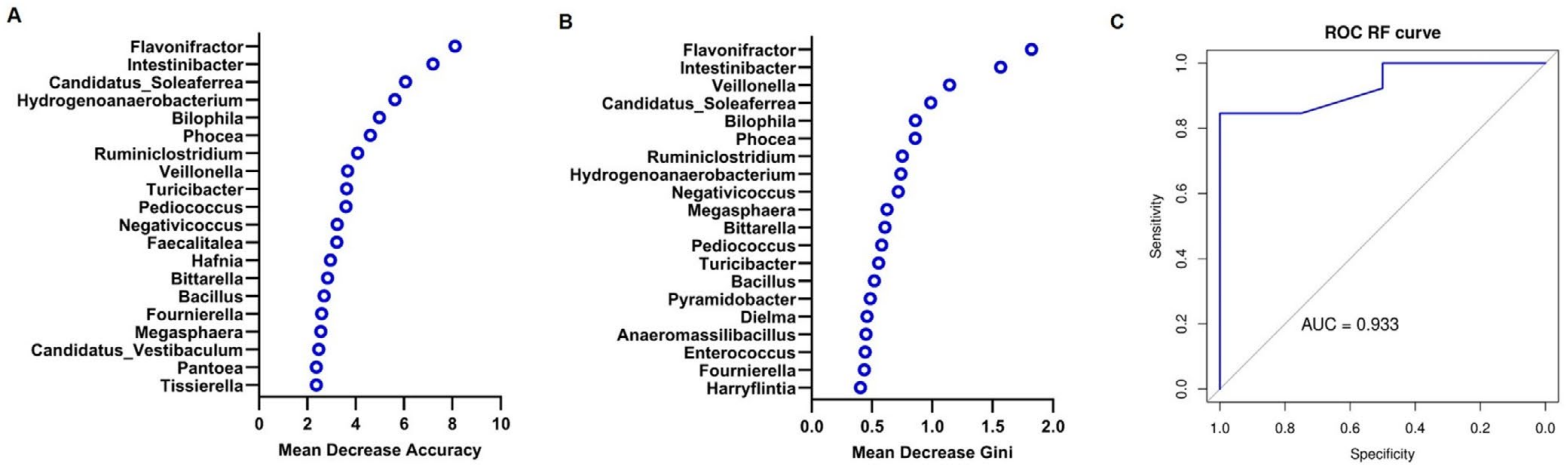
Random Forest model for PHC and PBTC classification at the genus level. Mean Decrease Accuracy measure of the top 20 most important genera in predicting the group. The higher the values, the more the genus is crucial for the prediction accuracy of the model (A). Mean Decrease Gini of the top 20 most important genera. The higher the value, the more the bacterium contributes to creating pure, homogeneous nodes within the decision trees (B). The performance of the model was assessed through an AUC analysis (C).

The application of a Random Forest classifier to predict bacterial genera best discriminating between PHC and PBTC patients revealed *Flavonifractor* and *Intestinibacter* as the two top contributors according to either Mean Decrease Accuracy (Figure 6A) and Mean Decrease Gini (Figure 6B). *Candidatus soleaferrea* also performed well in classifying the two groups, being respectively third and fourth according to the two above‐cited importance metrics. This model exhibited excellent performance, as revealed by the ROC curve in Figure 6C, showing an AUC of 0.933 (95% CI: 0.829–1.000), achieving a specificity of 100% while maintaining high sensitivity (85%).

### 2.6 Correlations Between Gut Microbiota and Hematological Parameters in PHC and PBTC Patients

**FIGURE 7:**
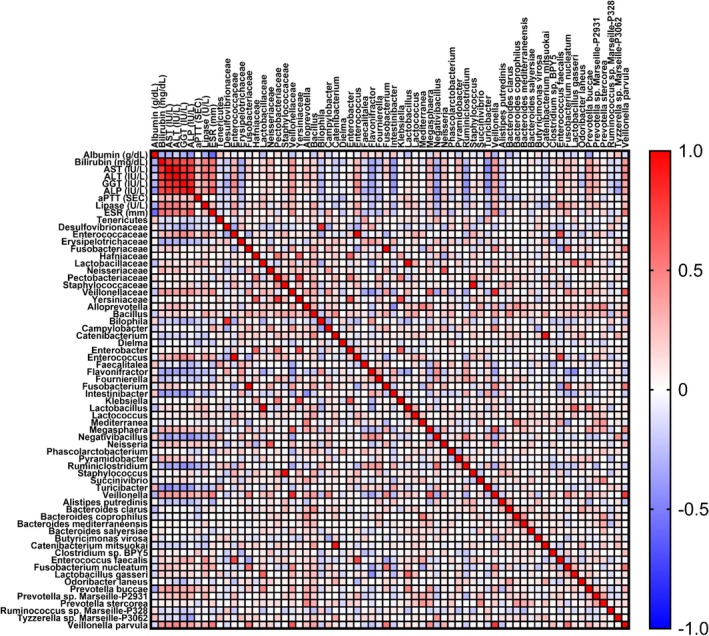
Spearman correlations of clinical parameters with intestinal bacteria at the phylum, family, genus and species levels that discriminate the two groups of patients (PHC and PBTC patients). The shades from blue (negative correlation) to red (positive correlation) indicate the value of the correlation coefficient R.

In order to investigate whether differences in gut microbiota composition could somehow be associated with changes observed in hematological parameters (Table 2), a Spearman correlation analysis was performed between bacteria differently represented in PHC and PBTC patients and altered blood parameters (Figure 7). Among the significant correlations observed, at the family level, *Enterococcaceae* showed a significant positive correlation with GGT (*r* = 0.46) and ALP (r = 0.41), together with Veillonellaceae that was positively correlated with bilirubin (*r* = 0.40), ALP (*r* = 0.43) and ESR (*r* = 0.45). At the genus level, *Enterococcus* exhibited a significant positive correlation with GGT (*r* = 0.46) and ALP (*r* = 0.40), while *Veillonella* was positively correlated with bilirubin (*r* = 0.41) and ESR (*r* = 0.50). On the contrary, *Intestinibacter* and *Ruminiclostridium* showed a negative correlation with GGT (*r* = −0.41 and *r* = −0.42, respectively), while *Negativibacillus* exhibited a significant negative correlation with ALP (*r* = −0.44), together with *Turicibacter* that was negatively correlated with AST (*r* = −0.46) and GGT (*r* = −0.42). At the species level, positive correlations emerged between.

*Enterococcus faecalis* and lipase (*r* = 0.45), between *Prevotella buccae* and AST (*r* = 0.40) and between *Veillonella parvula* and bilirubin (*r* = 0.40), AST (*r* = 0.44), ALT (*r* = 0.48) and ESR (*r* = 0.52).

## 3 Discussion and Conclusions

PC is a deadly malignancy, often diagnosed at late stages due to the lack of specific symptoms and reliable biomarkers. Research suggests that the gut microbiota plays a role in PC development and progression, which is why the first aim of the current study was to investigate gut microbial profiles discriminating PC patients from healthy controls. A 16S rRNA gene sequencing approach revealed substantial differences in alpha‐ and beta‐diversity and at the compositional level, indicating a condition of gut dysbiosis in PC patients. Reduced microbial evenness in PC patients, reflected in lower Shannon index values at the genus and species levels, suggests a less stable microbial ecosystem dominated by few taxa [24, 25, 26]. The reduced F/B ratio in PC patients aligns with findings in metabolic disorders and other malignancies, in which reduced F/B ratios are associated with inflammation and metabolic dysregulation [27]. At the phylum level, PC patients exhibited enrichment of Bacteroidetes, Proteobacteria and Fusobacteria, with depletion of Firmicutes. These changes suggest a shift toward pro‐inflammatory and barrier‐disrupting taxa, as Proteobacteria and Fusobacteria produce endotoxins like lipopolysaccharide (LPS), promoting tumorigenic environments [28, 29]. At the family level, the significant enrichment of Bacteroidaceae and Enterobacteriaceae in PC patients reflects the expansion of opportunistic pathogens capable of inducing inflammation. Conversely, the depletion of Ruminococcaceae and Lachnospiraceae suggests a loss of short‐chain fatty acids (SCFAs) producers critical for gut homeostasis [30]. This was also observed at the genus level, with the depletion of beneficial butyrate producers such as *Faecalibacterium*, *Ruminococcus* and *Coprococcus* and the increase in pro‐inflammatory and potentially pathogenic bacteria such as *Fusobacterium*, *Escherichia*, *Enterococcus* and *Veillonella* [15, 31, 32]. Another well‐documented genus enriched in PC patients is *Klebsiella*, which has been implicated in promoting tumorigenesis in epithelial cells [33]. Similarly, certain species within the *Campylobacter* genus, also enriched in PC patients, appear to produce genotoxins that contribute to carcinogenesis in the colorectal region [34]. Moreover, the increase observed in *Odoribacter* aligned with findings from previous studies, supporting a potential role in the pathophysiology of PC [35]. Interestingly, the genera *Lactobacillus* and *Bifidobacterium* were also found enriched in the fecal microbiota of PC patients, paralleling the findings by Kartal et al., in which these two genera were found more abundant in tumor pancreatic tissues than in healthy ones [17]. At the species level, *Fusobacterium nucleatum* and *Bacteroides fragilis* were found enriched in PC patients. These are well‐documented pro‐inflammatory and genotoxic taxa, implicated in promoting epithelial barrier disruption, immune evasion and tumor progression through mechanisms such as the production of genotoxins and inflammatory mediators [36, 37]. Similarly, *Enterococcus faecalis* and *Klebsiella pneumoniae* , which we found enriched in PC patients compared to healthy controls, are known for their roles in oxidative stress and inflammation, driven by the production of reactive oxygen species and bacterial endotoxins, which may exacerbate tumor‐associated inflammation [38, 39]. Additionally, multiple species within the *Lactobacillus* genus, including *Lactobacillus crispatus* , *Lactobacillus delbrueckii* , *Lactobacillus plantarum* , *Lactobacillus reuteri* and *Lactobacillus rhamnosus* , were found enriched in PC patients. Lactobacilli are typically associated with beneficial metabolic and immunomodulatory effects, although strain‐level differences can justify functional variations in metabolic potential and interaction with the environment [40]. Their enrichment in PC may reflect an adaptive response to the tumor‐altered microenvironment or interactions with host metabolic pathways that favor tumor progression, as suggested by Kartal et al. [41]. Two recent studies [42, 43], moreover, demonstrated a role for intra‐tumor lactobacilli in suppressing anti‐tumor immunity in PC, either in mice and in humans. Specifically, bacteria belonging to the genus *Lactobacillus* are able to metabolize dietary tryptophan to indoles which can, in turn, be sensed by the aryl hydrocarbon receptor expressed by tumor‐associated macrophages and create an immune‐suppressive environment [42]. Further taxa enriched in PC include *Porphyromonas gingivalis* and *Shigella dysenteriae* , both recognized for their potential to induce chronic inflammation and epithelial disruption [44, 45]. *Streptococcus anginosus* and *Streptococcus pyogenes* were also identified, supporting their possible roles in promoting inflammatory responses and immune modulation within the tumor microenvironment [46]. Notably, beneficial species such as *Coprococcus* sp. ART55/1, *Dialister* sp. S7D, *Roseburia intestinalis* , *Roseburia* sp. MC_37 and *Lachnospira pectinoschiza*, recognized for producing SCFAs and exerting anti‐inflammatory effects [26, 47, 48, 49, 50], were found significantly diminished in PC as compared to healthy controls. A striking reduction in *Methanobrevibacter smithii* was also observed in PC patients. This archaeon is pivotal for hydrogen metabolism in the gut and plays a synergistic role in optimizing microbial fermentation processes, suggesting its depletion could indicate broader metabolic imbalances in the PC microbiota [51, 52]. Interestingly, *Escherichia coli* was also less abundant in PC patients compared to CTRL. While *E. coli* has been associated with pro‐inflammatory and pathogenic roles under certain conditions, its reduction in this context could indicate complex shifts in microbial community dynamics and competition within the gut ecosystem [53].

Tumors located in the pancreatic head exhibited distinct clinical and biological characteristics compared to those located in the body or tail of the pancreas. We then sought to identify gut bacterial markers able to discriminate between these two subgroups and to investigate whether such microorganisms could be associated with the different clinical manifestations. In our cohort, PHC and PBTC patients showed distinct clinical presentations and biochemical profiles. The higher prevalence of jaundice in PHC patients is directly linked to bile duct obstruction caused by tumors in the pancreatic head. Similarly, the higher use of endoprostheses in PHC patients reflects the clinical necessity to alleviate biliary obstruction and associated complications [54]. Distinct biochemical profiles were observed between PHC and PBTC patients. PHC patients showed elevated bilirubin, AST, ALT, GGT, ALP and ESR levels, reflecting the systemic impact of obstructive jaundice and hepatic dysfunction [55]. This cholestasis condition may indirectly shape gut microbial communities. Altered bile flow and systemic cholestasis influence intestinal ecology both through direct antimicrobial activity of bile acids and through indirect stimulation of the production of antimicrobial peptides, which inhibit gut bacteria overgrowth [56, 57]. Furthermore, hyperbilirubinemia and cholestasis can impair nutrient absorption and energy balance, thereby modifying substrate availability for gut microbes [58]. Indeed, in our analysis, these abovementioned blood biomarkers also correlated with changes in gut microbiota. Noteworthy, in our study population, we also observed a positive correlation between Enterococcaceae (and its cognate genus *Enterococcus*) and the cholestasis markers GGT and ALP, and a positive association between *Enterococcus faecalis* and the pancreatic marker lipase; the three markers were found increased in PHC patients as compared to PBTC patients. Although correlation does not imply a cause‐effect relationship between two variables, previous studies describing an increase of these microorganisms in cirrhosis [59], chronic hepatitis C, hepatocellular carcinoma [60] and infantile cholestasis [61] support our findings and suggest an involvement of such bacteria in pathological conditions affecting the liver. By contrast, PBTC patients more often present without overt cholestasis and thus maintain a relatively preserved metabolic and nutritional status at diagnosis [62] as confirmed by higher red blood cell counts and albumin levels. When analyzing the compositional differences between the two subgroups of patients, PBTC showed enrichment of *Desulfovibrionaceae*, known producers of hydrogen sulfide, which can impair gut barrier function and promote systemic inflammation [63]. Moreover, genera such as *Ruminiclostridium*, *Ruminococcus*, *Anaeromassilibacillus*, *Bilophila, Flavonifractor* and *Intestinibacter* were significantly enriched in PBTC patients compared to PHC patients. These genera are predominantly associated with SCFAs production, particularly butyrate, which plays a critical role in maintaining intestinal homeostasis, regulating immune responses and suppressing inflammation [64, 65, 66, 67, 68, 69]. Interestingly, *Flavonifractor* and *Intestinibacter* were identified as the top two most influent bacterial genera in classifying PHC and PBTC patients, when a Random Forest algorithm was applied to discriminate the two groups.

On the contrary, *Fusobacterium*, *Veillonella*, *Enterobacter*, *Campylobacter*, *Enterococcus*, *Klebsiella* and *Lactobacillus* were significantly reduced in PBTC compared to PHC patients. The decrease in *Fusobacterium* levels observed in PBTC patients is worth noting, given the role of this genus in fostering tumor‐associated inflammation and immune evasion through mechanisms such as myeloid‐derived suppressor cell recruitment and T‐cell inhibition, particularly prominent in the localized inflammatory environment of the pancreatic head [33, 61]. Similarly, the lower abundance of *Veillonella* in PBTC patients may reflect differences in metabolic microenvironments, as this genus is known to metabolize lactate into SCFAs, which in turn influence immune responses and tumor metabolism [70]. *Enterobacter*, associated with endotoxin production and chronic inflammation through LPS‐mediated TLR4 signaling, was also found depleted in PBTC patients, suggesting a less robust inflammatory milieu [71, 72]. Similarly, the genus *Campylobacter* was less abundant in PBTC patients, possibly due to the distinct metabolic and bile acid profiles of the pancreatic body and tail [73]. Additionally, *Enterococcus* and *Klebsiella*, both implicated in epithelial barrier disruption and inflammation through reactive oxygen species (ROS) and LPS production, respectively, showed reduced levels in PBTC patients [38, 74]. Finally, *Lactobacillus*, traditionally regarded as beneficial, was enriched in PHC patients, potentially reflecting localized inflammation or bile acid interactions, whereas its depletion in PBTC patients may mean that tumors in the body and tail of the pancreas have different metabolic demands [75, 76]. At the species level, the pro‐inflammatory and immune‐modulating *Fusobacterium nucleatum* and *Prevotella stercorea*, were found enriched in PHC as compared to PBTC patients, suggesting a localized dysbiosis near the duodenum and bile duct [36, 77]. Conversely, the anti‐inflammatory and SCFA producer *Odoribacter laneus* , was significantly enriched in PBTC patients. This enrichment may indicate a more balanced gut microbiota in PBTC patients compared to the dysbiosis observed in PHC patients. The differences in gut microbial profiles between PBTC and PHC reveal the importance of stratifying PC patients by tumor localization when developing microbiota‐based interventions. Targeting *Fusobacterium* in PHC patients and promoting SCFA‐producing bacteria in PBTC patients may provide therapeutic benefits [78].

Overall, the current study confirms that a dysbiotic condition characterizes the gut microbial community of PC patients as compared to healthy subjects, supporting findings from previous studies [17, 35]. Most importantly, to the best of our knowledge, our study is the first to investigate the gut microbial differences between PHC and PBTC, whose distinctive clinical and molecular features have long been studied [79, 80, 81, 82]. The main objective of our exploratory study was to assess the existence of different bacterial profiles and, likely, different functional pathways between the two subgroups, which could help to better understand the biological differences between PC arising from the head or from the body‐tail, and hopefully, help to develop tailored interventional modalities taking into account the metabolic alterations characteristic of the two separate locations.

Moreover, the use of Random Forest to find out bacteria with the highest discriminatory power between the analyzed conditions could suggest new targets on which to focus for future clinical applications.

Of course, the current study presents some limitations. First of all, it is based on a single‐center cohort and lacks a validation dataset. PC is not as common as many other types of tumors, making it difficult to gather large patient cohorts for validation studies. However, the application of Random Forest, which uses the Out‐of‐bag validation, makes it less strictly necessary to have a separate validation dataset. Another limitation is the lack of a multivariate analysis for the control of confounding factors, such as medications or diet. Several drug classes known to alter gut composition were represented in our cohort. For example, among antihypertensives, ACE inhibitors have been linked to shifts in microbial composition and metabolites in hypertensive patients [83, 84]. For antidiabetic therapy, metformin causes changes in the composition of microbiota, such as a depletion of butyrate‐producing bacteria [85, 86]. Moreover, pancreatic enzyme replacement therapy and exocrine pancreatic insufficiency alter nutrient availability and microbial fermentation, representing additional potential biases [87, 88]. Dietary patterns are also known to shape the microbiota; for instance, Mediterranean or vegetarian diets are often associated with higher diversity and SCFA‐producing taxa [89, 90]. Finally, in the current study, the functional prediction and the putative mechanistic impact of the microbial shifts described have not been afforded. To strengthen the translational value of our findings, further studies integrating multi‐omics approaches would be desirable to unravel the intricate host‐microbiota interactions. In this regard, future directions could include: (i) prospective, multi‐cohort validation essential to confirm reproducibility and reduce overfitting; (ii) shotgun metagenomic sequencing that may provide higher taxonomic and functional resolution compared to 16S profiling, capturing strain‐level variation and metabolic pathways relevant to PC; (iii) integration with blood biomarkers (e.g., cytokines, metabolites) that could improve predictive performance and yield composite panels with higher robustness; (iv) functional validation of candidate taxa and metabolites in experimental models that would be crucial to move from correlative to mechanistic inference. Despite being preliminary, the findings described in the present work encourage future research to make gut microbiota a target for the development of prognostic tools and tailored therapeutic interventions, aimed at improving PC patients' clinical outcome.

## 4 Materials and Methods

### 4.1 Study Participants

PC patients were recruited between June 2019 and May 2024, while healthy subjects were enrolled between January 2018 and May 2024. Both groups were selected at the Fondazione IRCCS “Casa Sollievo della Sofferenza” Hospital in San Giovanni Rotondo, Italy, following standardized protocols for the collection, processing and preservation of biological samples. Ethical approval for the study was granted by the IRCCS “Casa Sollievo della Sofferenza” Hospital under Ethical Committee approval number N.184/CE and all participants provided written informed consent (IC). The study was conducted in accordance with the principles of Good Clinical Practice, the Declaration of Helsinki and in compliance with national legislation. Comprehensive information regarding gender, age, height, weight, body mass index (BMI), alcohol consumption, smoking status and dietary habits was collected. For PC patients, additional clinical characteristics and hematological parameters were assessed. All data were retrieved from hospital records and through direct patient interviews. Inclusion criteria for PC patients required a confirmed diagnosis at the time of enrollment, prior to the initiation of any cancer treatment. Patients were further stratified into two groups based on tumor location: pancreatic head cancer and pancreatic body‐tail cancer. Both patient groups underwent thorough clinical examinations, including assessments of basic anthropometric data and evaluations of blood and serum tests. Exclusion criteria included any antibiotic use within the 3 months prior to enrollment, applied to both PC patients and healthy controls.

### 4.2 Laboratory Test Analysis

Data on blood and serum/plasma analyses for PHC and PBTC patients were manually retrieved from medical records. Parameters evaluated included serum glucose, total protein, albumin, bilirubin, transaminases, gamma‐glutamyl transferase (GGT), alkaline phosphatase (ALP), pancreatic amylase, lipase, creatinine, C‐reactive protein (CRP), carcinoembryonic antigen (CEA) and carbohydrate antigen 19‐9 (CA19‐9). Hematological assessments also encompassed erythrocyte sedimentation rate (ESR), complete blood counts (hemoglobin, erythrocyte, leukocyte, neutrophil and platelet counts), as well as coagulation profiles such as prothrombin time (PT) and partial thromboplastin time (PTT). All analyses were performed in compliance with clinical protocols at certified laboratories at the Fondazione IRCCS “Casa Sollievo della Sofferenza” Hospital, San Giovanni Rotondo, Italy.

### 4.3 Sample Collection and DNA Extraction

Fecal samples were collected from each participant in a tube and then stored at −80°C for preservation until further processing. Genomic DNA was extracted from human stool samples using the QIAamp Fast DNA Stool Mini Kit (Qiagen, Milan, Italy, cat. no. 51604), in accordance with the manufacturer's protocol. To enhance the proportion of microbial DNA relative to human DNA, difficult‐to‐lyse cells, including gram‐positive bacteria, were lysed by heating the fecal suspension at 90°C for 5 min. Following the isolation protocol, the DNA yield and purity were assessed and samples were subsequently stored at −30°C until use.

### 4.4 Next‐Generation Sequencing and Analysis of Bacterial 16S rRNA Gene

A total of 12.5 ng of DNA, extracted following the protocol described above, was used for the amplification of the V3‐V4 region of the 16S rRNA gene. The amplification was performed using the KAPA HiFi HotStart Ready Mix (Roche Diagnostics, Milan, Italy, Cat. No. 07958935001) and primers described by Klindworth [91], with Illumina adapters (underlined) incorporated: forward primer 5′‐TCGTCGGCAGCGTCAGATGTGTATAAGAGACAGCCTACGGGNGGCWGCAG and reverse primer 5′‐GTCTCGTGGGCTCGGAGATGTGTATAAGAGACAGGACTACHVGGGTATCTAATCC. Barcoding of the samples was carried out with the Nextera XT Index Kit (Illumina, Milan, Italy, cat. no. FC‐131‐1002), following previously established protocols [92]. Subsequently, libraries were purified, quantified using a Qubit Flex Fluorometer (Thermo Scientific, Milan, Italy), pooled and sequenced in paired‐end mode (2 × 300 cycles) on an Illumina MiSeq platform (San Diego, CA, USA). The resulting FASTQ files were submitted to ArrayExpress under accession code E‐MTAB‐14783. Sequences were de‐multiplexed and analyzed using GAIA 2.0 software for 16S Metagenomics (Sequentia Biotech, Barcelona, Spain, 2019). Quality control of read pairs—including trimming, clipping and adapter removal—was performed using FastQC and BBDuk. Taxonomic profiling was achieved by mapping reads with BWA‐MEM against the NCBI 16S reference database.

### 4.5 Statistical Methods

The anthropometric, lifestyle and clinical characteristics of the study participants were summarized using appropriate statistical measures: means and standard deviations for normally distributed continuous variables, medians and interquartile ranges for non‐normally distributed continuous variables and absolute frequencies with corresponding percentages for categorical variables. Group comparisons were conducted using statistical tests tailored to the data type and distribution, including the two‐sample *t*‐test for normally distributed continuous variables, the Mann–Whitney *U* test and Fisher's exact test for non‐parametric continuous variables and the Chi‐Square test with Yates' continuity correction for categorical variables. Data were considered statistically significant at three levels: *p* < 0.05 (*), *p* < 0.01 (**) and *p* < 0.001 (***).

Alpha‐diversity metrics Shannon index and Chao1 index were calculated by GAIA 2.0 software and represented as box plots using GraphPad Prism v9.3.1.

As for beta‐diversity, Principal Component Analyses and PERMANOVA tests on Bray–Curtis distances (calculated by GAIA 2.0 software) were performed in R (version 4.3.2) using the packages vegan, ape, and ggplot2 for statistics, ordination, and data visualization.

Stacked bar charts were used to show the gut microbiota composition (i.e., mean relative abundance %) at phylum, family, genus and species levels, comparing PC versus CTRL groups as well as PHC versus PBTC subgroups. Differential analysis of taxonomic data was conducted using the DESeq2 statistical framework, with results deemed significant when the *p* < 0.05 and the false discovery rate (FDR) < 0.05. A Random Forest classification model was built in R (v 4.3.2) to discriminate between sample groups, on differential abundant genera, based on their natural logarithm‐transformed bacterial absolute abundance profiles [log(*x* + 1)]. The dataset was split into a training (70%) and a test (30%) set using the caret package. Using the randomForest package, a model was constructed with 500 trees. Performance was evaluated by calculating the area under the curve (AUC‐ROC), sensitivity, specificity, and the 95% confidence interval for the AUC using the pROC package. The most influential genera were identified from the model's variable importance scores. Correlations between patients' clinical parameters and microbiota data were assessed using GraphPad Prism v9.3.1, applying Spearman's rank correlation coefficient due to the non‐parametric nature of the data. All statistical computations were carried out using R software and GraphPad Prism, ensuring robust adjustments for multiple comparisons where applicable.

## Author Contributions

**Annacandida Villani:** data curation (equal), investigation (lead), methodology (equal), writing – original draft (lead). **Gandino Mencarelli:** data curation (equal), investigation (equal), software (lead), writing – original draft (supporting). **Giovanna Cocomazzi:** data curation (supporting), investigation (supporting), methodology (supporting). **Elena Binda:** data curation (supporting), formal analysis (supporting), supervision (equal), validation (equal), writing – review and editing (equal). **Edy Virgili:** methodology (supporting), visualization (supporting), writing – original draft (supporting). **Tiziana Pia Latiano:** resources (supporting), visualization (supporting). **Evaristo Maiello:** resources (supporting), validation (equal), visualization (equal). **Viviana Contu:** data curation (equal), methodology (equal), resources (supporting), supervision (equal), visualization (equal), writing – original draft (equal). **Francesco Perri:** methodology (equal), resources (equal), validation (equal), visualization (equal). **Concetta Panebianco:** data curation (equal), investigation (equal), methodology (equal), supervision (equal), writing – original draft (equal). **Valerio Pazienza:** conceptualization (lead), formal analysis (equal), funding acquisition (lead), investigation (equal), methodology (supporting), project administration (lead), resources (equal), supervision (equal), validation (equal), visualization (equal), writing – review and editing (equal).

## Funding

The research leading to these results received funding from the Italian Association for Cancer Research (AIRC) under IG 2019‐ID. 23006 project—P.I. Pazienza Valerio. This work was also supported by the Italian Ministry of Health through the Ricerca Corrente Program RC2022‐2024 granted to V.P. and by the 5x1000 voluntary contribution to “Fondazione Casa Sollievo della Sofferenza IRCCS”.

## Ethics Statement

Ethical approval was obtained from the IRCCS “Casa Sollievo della Sofferenza” Hospital, under the Ethical Committee approval number N.184/CE.

## Conflicts of Interest

The authors declare no conflicts of interest.

## Supporting information


**Table S1:** Complete list of bacterial taxa differentially represented between PC and CTRL.

**Table S2:** Complete list of bacterial taxa differentially represented between PHC and PBTC patients.

## Data Availability

FASTQ files containing 16S rRNA gene sequencing raw data were deposited in the repository ArrayExpress under the accession code E‐MTAB‐14783.
