# Supplementary material for: Comparative Analysis of Fecal Microbiota in Healthy Controls and Pancreatic Cancer Patients: A Focus on Tumor Localization Differences in Pancreatic Head and Body–Tail
Source: Cancer Med. 2025 Dec 12;14(24):e71450. doi: 10.1002/cam4.71450 (PMC12700705; doi:10.1002/cam4.71450)
Supplement: Supplementary file 1 — Table S1: Complete list of bacterial taxa differentially represented between PC and CTRL. [file CAM4-14-e71450-s001.docx]

**Supplementary Table 1.** Complete list of bacterial taxa differentially represented between PC and CTRL.

|  | **Relative abundance in PC (%)** | | | **Relative abundance in CTRL (%)** | | **logFC.deseq2** | | **P-value** | | **FDR** |
| --- | --- | --- | --- | --- | --- | --- | --- | --- | --- | --- |
| **Increased in PC** |  | | |  | |  | |  | |  |
| Bacteroidetes | 46.97 | | | 33.24 | | 0.61 | | 1.07x10⁻⁶ | | 1.16x10⁻⁵ |
| Fusobacteria | 0.22 | | | 0.02 | | 3.82 | | 8.71x${10}^{-14}$ | | 1.87${10}^{-12}$ |
| Proteobacteria | 9.94 | | | 3.17 | | 1.76 | | 2.46${x10}^{-16}$ | | 1.06${x10}^{-14}$ |
| Synergistetes | 0.47 | | | 0.09 | | 2.11 | | 5.52${x10}^{-5}$ | | 0.0003 |
| *Bacteroidaceae* | 28.98 | | | 20.43 | | 0.92 | | 2.41${x10}^{-5}$ | | 0.0001 |
| *Enterobacteriaceae* | 4.16 | | | 0.81 | | 2.61 | | 8.43${x10}^{-10}$ | | 1.44${x10}^{-8}$ |
| *Enterococcaceae* | 0.61 | | | 0.02 | | 4.11 | | 3.94${x10}^{-28}$ | | 1.42${x10}^{-25}$ |
| *Fusobacteriaceae* | 0.21 | | | 0.02 | | 3.52 | | 6.53${x10}^{-11}$ | | 1.50${x10}^{-9}$ |
| *Lactobacillaceae* | 1.66 | | | 0.09 | | 3.11 | | 4.75${x10}^{-14}$ | | 1.55${x10}^{-12}$ |
| *Odoribacteraceae* | 1.64 | | | 0.73 | | 1.42 | | 5.39${x10}^{-5}$ | | 0.0003 |
| *Porphyromonadaceae* | 3.82 | | | 2.53 | | 1.14 | | 4.46${x10}^{-5}$ | | 0.0002 |
| *Pseudomonadaceae* | 0.11 | | | 0.007 | | 2.44 | | 8.70${x10}^{-15}$ | | 3.13${x10}^{-13}$ |
| *Streptococcaceae* | 0.13 | | | 0.38 | | 1.88 | | 2.37${x10}^{-7}$ | | 2.51x10⁻⁶ |
| *Succinivibrionaceae* | 0.31 | | | 0.02 | | 2.83 | | 0.0016 | | 0.006 |
| *Sutterellaceae* | 2.81 | | | 0.97 | | 1.95 | | 7.93${x10}^{-10}$ | | 1.42${x10}^{-8}$ |
| *Synergistaceae* | 0.46 | | | 0.09 | | 2.36 | | 6.15x10⁻⁶ | | 4.34${x10}^{-5}$ |
| *Veillonellaceae* | 3.64 | | | 1.80 | | 1.35 | | 0.0005 | | 0.002 |
| *Victivallaceae* | 0.20 | | | 0.07 | | 1.57 | | 0.014 | | 0.045 |
| *Acidaminococcus* | 0.63 | | | 0.24 | | 2.95 | | 5.66x10⁻⁶ | | 4.70${x10}^{-5}$ |
| *Anaerostipes* | 0.37 | | | 0.11 | | 1.87 | | 2.00${x10}^{-5}$ | | 0.0001 |
| *Bacteroides* | 28.74 | | | 20.24 | | 1.32 | | 6.66${x10}^{-9}$ | | 1.05${x10}^{-7}$ |
| *Bifidobacterium* | 1.51 | | | 1.25 | | 1.08 | | 0.009 | | 0.03 |
| *Butyricimonas* | 0.77 | | | 0.33 | | 1.25 | | 0.003 | | 0.013 |
| *Campylobacter* | 0.01 | | | 0.002 | | 3.63 | | 3.97${x10}^{-17}$ | | 2.83${x10}^{-15}$ |
| *Coprobacter* | 0.21 | | | 0.06 | | 2.12 | | 7.62${x10}^{-5}$ | | 0.0004 |
| *Enterococcus* | 0.59 | | | 0.01 | | 5.66 | | 1.11${x10}^{-33}$ | | 3.4${x10}^{-31}$ |
| *Escherichia* | 0.27 | | | 0.09 | | 1.78 | | 5.30${x10}^{-5}$ | | 0.0003 |
| *Fusobacterium* | 0.19 | | | 0.02 | | 4.66 | | 2.35${x10}^{-18}$ | | 1.825${x10}^{-16}$ |
| *Klebsiella* | 0.91 | | | 0.02 | | 6.90 | | 4.52${x10}^{-37}$ | | 4.19${x10}^{-34}$ |
| *Lactobacillus* | 1.62 | | | 0.08 | | 3.26 | | 4.93${x10}^{-15}$ | | 2.41${x10}^{-13}$ |
| *Megasphaera* | 0.14 | | | 0.10 | | 6.09 | | 6.92${x10}^{-29}$ | | 1.60${x10}^{-26}$ |
| *Neisseria* | 0.005 | | | 0.0005 | | 3.04 | | 2.75${x10}^{-5}$ | | 0.0002 |
| *Odoribacter* | 0.85 | | | 0.38 | | 1.61 | | 1.22${x10}^{-5}$ | | 9.42${x10}^{-5}$ |
| *Parabacteroides* | 2.87 | | | 1.80 | | 1.63 | | 5.45${x10}^{-7}$ | | 6.02x10⁻⁶ |
| *Prevotellamassilia* | 0.28 | | | 0.004 | | 1.59 | | 0.006 | | 0.020 |
| *Pseudomonas* | 0.11 | | | 0.006 | | 2.97 | | 1.70${x10}^{-18}$ | | 1.45${x10}^{-16}$ |
| *Pyramidobacter* | 0.42 | | | 0.008 | | 7.47 | | 2.79${x10}^{-19}$ | | 2.88${x10}^{-17}$ |
| *Shigella* | 0.02 | | | 0.004 | | 3.10 | | 1.03${x10}^{-9}$ | | 2.00${x10}^{-8}$ |
| *Staphylococcus* | | | 0.01 | 0.002 | | 3.35 | | 2.42${x10}^{-9}$ | | 4.41${x10}^{-8}$ |
| *Streptococcus* | | | 1.04 | 0.36 | | 1.91 | | 5.85${x10}^{-7}$ | | 6.39x10⁻⁶ |
| *Sutterella* | | | 1.64 | 0.47 | | 1.94 | | 0.0001 | | 0.0009 |
| *Tyzzerella* | | | 0.30 | 0.21 | | 1.00 | | 0.005 | | 0.017 |
| *Veillonella* | | | 2.70 | 0.14 | | 5.62 | | 1.00${x10}^{-34}$ | | 4.67${x10}^{-32}$ |
| *Victivallis* | | | 0.16 | 0.05 | | 1.96 | | 0.002 | | 0.008 |
| *[Eubacterium] hallii* | | | 0.20 | 0.02 | | 3.93 | | 1.04${x10}^{-21}$ | | 2.00${x10}^{-19}$ |
| *Acidaminococcus intestini* | | | 0.42 | 0.18 | | 2.76 | | 0.001 | | 0.007 |
| *Anaerostipes hadrus* | | | 0.21 | 0.04 | | 2.37 | | 3.16${x10}^{-6}$ | | 3.02${x10}^{-5}$ |
| *Bacteroides caccae* | | | 1.32 | 0.53 | | 1.92 | | 0.0001 | | 0.0006 |
| *Bacteroides dorei* | | | 1.08 | 0.26 | | 3.69 | | 3.03${x10}^{-11}$ | | 8.68${x10}^{-10}$ |
| *Bacteroides fragilis* | | | 0.75 | 0.20 | | 2.72 | | 1.78${x10}^{-5}$ | | 0.0001 |
| *Bacteroides ovatus* | | | 1.12 | 0.33 | | 2.51 | | 2.99${x10}^{-8}$ | | 4.68${x10}^{-7}$ |
| *Bacteroides thetaiotaomicron* | | | 0.54 | 0.20 | | 1.86 | | 2.19${x10}^{-5}$ | | 0.0001 |
| *Bacteroides uniformis* | | | 0.68 | 0.49 | | 0.93 | | 0.016 | | 0.047 |
| *Barnesiella* sp. S496 | | | 0.11 | 0.05 | | 1.79 | | 0.007 | | 0.024 |
| *Blautia obeum* | | | 0.31 | 0.11 | | 1.88 | | 2.26${x10}^{-8}$ | | 3.60${x10}^{-7}$ |
| *Butyricimonas* sp. S479 | | | 0.12 | 0.01 | | 2.42 | | 0.001 | | 0.006 |
| *Clostridioides difficile* | | | 0.01 | 0.002 | | 2.19 | | 7.96${x10}^{-5}$ | | 0.0005 |
| *Clostridium perfringens* | | | 0.02 | 0.004 | | 1.66 | | 0.001 | | 0.004 |
| *Coprobacter fastidiosus* | | | 0.12 | 0.03 | | 2.46 | | 0.001 | | 0.005 |
| *Enterococcus faecalis* | | | 0.29 | 0.004 | | 6.89 | | 1.97${x10}^{-20}$ | | 2.82${x10}^{-18}$ |
| *Escherichia coli* | | | 0.15 | 0.49 | | 2.24 | | 1.30${x10}^{-7}$ | | 1.73${x10}^{-6}$ |
| *Fusobacterium nucleatum* | | | 0.06 | 0.0004 | | 6.20 | | 2.56${x10}^{-5}$ | | 0.0001 |
| *Klebsiella pneumoniae* | | | 0.18 | 0.006 | | 6.87 | | 7.72${x10}^{-25}$ | | 2.21${x10}^{-22}$ |
| *Lactobacillus crispatus* | | | 0.002 | 0.0001 | | 4.75 | | 3.58${x10}^{-6}$ | | 3.38${x10}^{-5}$ |
| *Lactobacillus plantarum* | | | 0.002 | 0.0002 | | 3.33 | | 0.0001 | | 0.0009 |
| *Lactobacillus reuteri* | | | 0.004 | 0.002 | | 4.50 | | 7.82${x10}^{-10}$ | | 1.77${x10}^{-8}$ |
| *Lactobacillus rhamnosus* | | | 0.002 | 3,24${x10}^{-6}$ | | 3.38 | | 0.005 | | 0.018 |
| *Odoribacter splanchnicus* | | | 0.53 | 0.25 | | 1.49 | | 0.0007 | | 0.003 |
| *Oscilibacter massiliensis* | | | 0.25 | 0.21 | | 1.13 | | 0.009 | | 0.03 |
| *Oscillibacter* sp. G2 | | | 0.16 | 0.05 | | 2.28 | | 2.66${x10}^{-5}$ | | 0.0002 |
| *Parabacteroides distasonis* | | | 0.74 | 0.37 | | 1.79 | | 1.11${x10}^{-5}$ | | 9.3${x10}^{-5}$ |
| *Parasutterella excrementihominis* | | | 0.78 | 0.17 | | 3.07 | | 1.31${x10}^{-6}$ | | 1.37${x10}^{-5}$ |
| *Porphyromonas gingivalis* | | | 0.0005 | 0.0002 | | 1.47 | | 0.004 | | 0.015 |
| *Prevotella* sp. DJF_RP53 | | | 0.47 | 0.20 | | 5.15 | | 1.36${x10}^{-10}$ | | 3.64${x10}^{-9}$ |
| *Prevotella stercorea* | | | 0.18 | 0.03 | | 4.84 | | 8.14${x10}^{-9}$ | | 1.45${x10}^{-7}$ |
| *Prevotellamassilia timonensis* | | | 0.25 | 0.0006 | | 3.50 | | 1.05${x10}^{-6}$ | | 1.12${x10}^{-5}$ |
| *Pyramidobacter piscolens* | | | 0.39 | 0.007 | | 7.13 | | 1.21${x10}^{-13}$ | | 5.47${x10}^{-12}$ |
| *Roseburia hominis* | | | 0.09 | 0.05 | | 1.18 | | 0.011 | | 0.034 |
| *Shigella dysenteriae* | | | 0.009 | 0.0007 | | 4.60 | | 5.31${x10}^{-16}$ | | 3.80${x10}^{-14}$ |
| *Streptococcus anginosus* | | 0.02 | | | 0.001 | | 4.88 | | 6.24${x10}^{-9}$ | 1.17${x10}^{-7}$ |
| *Streptococcus pyogenes* | | 0.0004 | | | 8.11${x10}^{-5}$ | | 1.99 | | 0.017 | 0.048 |
| *Sutterella* sp. 252 | | 0.20 | | | 0.04 | | 2.70 | | 0.0015 | 0.006 |
| *Sutterella* sp. YIT 12072 | | 0.20 | | | 0.01 | | 9.55 | | 4.18${x10}^{-14}$ | 2.11${x10}^{-12}$ |
| *Sutterella wadsworthensis* | | 0.85 | | | 0.20 | | 2.61 | | 0.0003 | 0.0016 |
| *Veillonella parvula* | | 0.11 | | | 0.02 | | 2.52 | | 0.001 | 0.005 |

|  | **Relative abundance in PC (%)** | **Relative abundance in CTRL(%)** | **logFC.deseq2** | **P-value** | **FDR** |
| --- | --- | --- | --- | --- | --- |
| **Decreased in PC** |  |  |  |  |  |
| Euryarchaeota | 0.01 | 0.14 | -3.66 | 2.16${x10}^{-5}$ | 0.0001 |
| Firmicutes | 36.30 | 56.08 | -0.59 | 1.23${x10}^{-7}$ | 1.76x10⁻⁶ |
| *Christensenellaceae* | 0.09 | 0.32 | -1.89 | 6.5${x10}^{-7}$ | 6x10⁻⁶ |
| *Eggerthellaceae* | 0.07 | 0.19 | -0.75 | 0.016 | 0.049 |
| *Erysipelotrichaceae* | 0.38 | 0.60 | -1.09 | 0.004 | 0.017 |
| *Eubacteriaceae* | 1.79 | 3.57 | -1.08 | 3.59x10⁻⁶ | 2.69${x10}^{-5}$ |
| *Lachnospiraceae* | 6.90 | 11.08 | -0.62 | 0.001 | 0.007 |
| *Methanobacteriaceae* | 0.005 | 0.14 | -4.40 | 1.83${x10}^{-8}$ | 2.63${x10}^{-7}$ |
| *Ruminococcaceae* | 8.58 | 20.48 | -1.07 | 6.17${x10}^{-7}$ | 5.85x10⁻⁶ |
| *Selenomonadaceae* | 0.22 | 0.72 | -2.74 | 4.32${x10}^{-10}$ | 8.63${x10}^{-9}$ |
| *Butyricicoccus* | 0.01 | 0.28 | -1.13 | 0.0001 | 0.0008 |
| *Christensenella* | 0.04 | 0.16 | -1.02 | 0.004 | 0.016 |
| *Coprococcus* | 0.22 | 0.10 | -2.01 | 6.11${x10}^{-7}$ | 6.6x10⁻⁶ |
| *Eubacterium* | 1.57 | 2.77 | -0.82 | 0.001 | 0.005 |
| *Faecalibacterium* | 4.53 | 11.09 | -0.84 | 0.003 | 0.012 |
| *Megamonas* | 0.18 | 0.57 | -2.79 | 0.002 | 0.010 |
| *Methanobrevibacter* | 0.005 | 0.14 | -4.15 | 2.42${x10}^{-7}$ | 2.96x10⁻⁶ |
| *Negativibacillus* | 0.03 | 0.14 | -1.40 | 0.001 | 0.008 |
| *Ruminiclostridium* | 0.15 | 0.49 | -1.42 | 1.36x10⁻⁶ | 1.35${x10}^{-5}$ |
| *Ruminococcus* | 2.23 | 5.16 | -0.74 | 0.011 | 0.036 |
| *Sporobacter* | 0.06 | 0.16 | -1.01 | 0.008 | 0.026 |
| *[Eubacterium] eligens* | 0.57 | 1.50 | -1.64 | 0.0006 | 0.003 |
| *[Eubacterium] siraeum* | 0.06 | 0.25 | -1.81 | 0.009 | 0.03 |
| *Bacteroides* sp. Marseille-P3108 | 0.14 | 0.17 | -1.24 | 0.001 | 0.004 |
| *Coprococcus eutactus* | 0.002 | 0.12 | -2.36 | 7.77${x10}^{-5}$ | 0.0005 |
| *Coprococcus* sp. ART55/1 | 0.05 | 0.44 | -3.36 | 3.73${x10}^{-7}$ | 4.45x10⁻⁶ |
| *Dialister* sp. S7D | 0.03 | 0.41 | -3.86 | 2.41${x10}^{-5}$ | 0.0001 |
| *Lachnospira pectinoschiza* | 0.11 | 0.25 | -1.58 | 0.001 | 0.005 |
| *Methanobrevibacter smithii* | 0.004 | 0.11 | -4.14 | 5.76${x10}^{-7}$ | 6.56x10⁻⁶ |
| *Roseburia intestinalis* | 0.11 | 0.53 | -2.08 | 0.0003 | 0.001 |
| *Roseburia* sp. MC_37 | 0.08 | 0.34 | -2.02 | 0.0006 | 0.002 |
