# Supplementary material for: Comparative Analysis of Fecal Microbiota in Healthy Controls and Pancreatic Cancer Patients: A Focus on Tumor Localization Differences in Pancreatic Head and Body–Tail
Source: Cancer Med. 2025 Dec 12;14(24):e71450. doi: 10.1002/cam4.71450 (PMC12700705; doi:10.1002/cam4.71450)
Supplement: Supplementary file 2 — Table S2: Complete list of bacterial taxa differentially represented between PHC and PBTC patients. [file CAM4-14-e71450-s002.docx]

**Supplementary Table 2**. Complete list of bacterial taxa differentially represented between PHC and PBTC patients.

|  | **Relative abundance in PBTC(%)** | **Relative abundance in PHC(%)** | **logFC.deseq2** | **P-value** | **FDR** |
| --- | --- | --- | --- | --- | --- |
| **Increased in PBTC** |  |  |  |  |  |
| Tenericutes | 1.17 | 0.07 | 5.75 | 4.14${x10}^{-24}$ | 1.74${x10}^{-22}$ |
| *Desulfovibrionaceae* | 0.70 | 0.28 | 1.38 | 0.002 | 0.049 |
| *Erysipelotrichaceae* | 0.51 | 0.30 | 1.45 | 0.0007 | 0.016 |
| *Anaeromassilibacillus* | 0.01 | 0.005 | 1.41 | 0.002 | 0.017 |
| *Bilophila* | 0.41 | 0.13 | 2.02 | 0.0002 | 0.003 |
| *Dielma* | 0.03 | 0.002 | 3.63 | 0.0007 | 0.007 |
| *Faecalitalea* | 0.08 | 0.005 | 4.37 | 3.72x10⁻⁶ | 0.0001 |
| *Flavonifractor* | 0.20 | 0.05 | 1.84 | 0.0001 | 0.002 |
| *Fournierella* | 0.02 | 0.001 | 0.85 | 0.003 | 0.024 |
| *Intestinibacter* | 0.06 | 0.01 | 2.72 | 0.001 | 0.013 |
| *Mediterranea* | 0.16 | 0.002 | 2.67 | 3.11${x10}^{-5}$ | 0.0006 |
| *Negativibacillus* | 0.04 | 0.02 | 1.51 | 0.002 | 0.017 |
| *Phascolarctobacterium* | 0.49 | 0.19 | 2.57 | 0.001 | 0.014 |
| *Ruminiclostridium* | 0.27 | 0.08 | 1.43 | 5.57${x10}^{-5}$ | 0.001 |
| *Turicibacter* | 0.01 | 0.001 | 3.89 | 5.22${x10}^{-5}$ | 0.0009 |
| *Alistipes putredinis* | 0.18 | 0.05 | 2.24 | 0.0005 | 0.046 |
| *Bacteroides mediterraneensis* | 0.75 | 0.10 | 3.81 | 1.68${x10}^{-5}$ | 0.003 |
| *Butyricimonas virosa* | 0.20 | 0.03 | 3.07 | 0.0001 | 0.017 |
| *Odoribacter laneus* | 0.32 | 0.0005 | 5.54 | 0.0001 | 0.020 |
| *Ruminococcus* sp. Marseille-P328 | 0.10 | 0.01 | 11.11 | 2.41${x10}^{-5}$ | 0.005 |
| *Tyzzerella* sp. Marseille-P3062 | 0.10 | 0.004 | 4.86 | 1.22${x10}^{-7}$ | 4.92${x10}^{-5}$ |

|  | **Relative abundance in PBTC(%)** | **Relative abundance in PHC(%)** | **logFC.deseq2** | **P-value** | **FDR** |
| --- | --- | --- | --- | --- | --- |
| **Decreased in PBTC** |  |  |  |  |  |
| *Enterococcaceae* | 0.11 | 0.91 | -2.50 | 8.54${x10}^{-11}$ | 2.15${x10}^{-8}$ |
| *Fusobacteriaceae* | 0.05 | 0.31 | -1.80 | 0.0009 | 0.019 |
| *Hafniaceae* | 0.003 | 0.08 | -1.95 | 0.0003 | 0.008 |
| *Lactobacillaceae* | 0.30 | 2.47 | -3.17 | 8.76${x10}^{-10}$ | 1.1${x10}^{-7}$ |
| *Neisseriaceae* | 0.002 | 0.01 | -3.03 | 7.02${x10}^{-7}$ | 5.05${x10}^{-5}$ |
| *Pectobacteriaceae* | 0.006 | 0.05 | -1.46 | 0.0003 | 0.009 |
| *Staphylococcaceae* | 0.003 | 0.02 | -2.04 | 0.0002 | 0.007 |
| *Veillonellaceae* | 0.97 | 5.24 | -2.46 | 4.51${x10}^{-10}$ | 7.55${x10}^{-8}$ |
| *Yersiniaceae* | 0.02 | 0.06 | -1.34 | 0.0004 | 0.011 |
| *Alloprevotella* | 0.004 | 0.06 | -2.29 | 1.46${x10}^{-5}$ | 0.0004 |
| *Bacillus* | 0.01 | 0.02 | -1.39 | 5.77${x10}^{-5}$ | 0.0010 |
| *Campylobacter* | 0.008 | 0.02 | -1.51 | 0.0008 | 0.008 |
| *Catenibacterium* | 0.03 | 0.20 | -7.24 | 1.32${x10}^{-9}$ | 1.5${x10}^{-7}$ |
| *Enterobacter* | 0.03 | 0.08 | -1.53 | 0.0003 | 0.0038 |
| *Enterococcus* | 0.11 | 0.88 | -4.06 | 3.91${x10}^{-15}$ | 1.11${x10}^{-12}$ |
| *Fusobacterium* | 0.02 | 0.29 | -1.87 | 0.0009 | 0.0089 |
| *Klebsiella* | 0.23 | 1.32 | -3.1 | 1.98${x10}^{-7}$ | 1.13${x10}^{-5}$ |
| *Lactobacillus* | 0.29 | 2.42 | -2.22 | 2.34x10⁻⁶ | 0.0001 |
| *Lactococcus* | 0.004 | 0.03 | -2.34 | 2.73${x10}^{-5}$ | 0.0006 |
| *Megasphaera* | 0.02 | 0.22 | -4.17 | 2.54${x10}^{-10}$ | 4.82${x10}^{-8}$ |
| *Neisseria* | 0.0007 | 0.007 | -3.65 | 1.18${x10}^{-5}$ | 0.0003 |
| *Pyramidobacter* | 0.24 | 0.53 | -4.41 | 1.67${x10}^{-8}$ | 1.36x10⁻⁶ |
| *Staphylococcus* | 0.002 | 0.02 | -2.19 | 0.0004 | 0.005 |
| *Succinivibrio* | 0.0008 | 0.48 | -3.11 | 0.005 | 0.03 |
| *Veillonella* | 0.52 | 4.01 | -3.09 | 4.44${x10}^{-10}$ | 6.32${x10}^{-8}$ |
| *Bacteroides clarus* | 0.01 | 0.11 | -2.22 | 3.78${x10}^{-5}$ | 0.006 |
| *Bacteroides coprophilus* | 0.13 | 0.52 | -5.45 | 2.28${x10}^{-9}$ | 2.51x10⁻⁶ |
| *Bacteroides salyersiae* | 0.02 | 0.19 | -3.23 | 0.0004 | 0.036 |
| *Catenibacterium mitsuokai* | 0.03 | 0.17 | -7.57 | 1.66${x10}^{-8}$ | 1.06${x10}^{-5}$ |
| *Clostridium* sp. BPY5 | 0.07 | 0.25 | -2.45 | 5.25x10⁻⁶ | 0.0013 |
| *Enterococcus faecalis* | 0.03 | 0.44 | -4.42 | 1.47${x10}^{-9}$ | 1.89x10⁻⁶ |
| *Fusobacterium nucleatum* | 0.0002 | 0.09 | -7.44 | 1.79${x10}^{-7}$ | 6.90${x10}^{-5}$ |
| *Lactobacillus gasseri* | 0.03 | 0.05 | -3.34 | 0.0005 | 0.044 |
| *Prevotella buccae* | 0.0009 | 0.12 | -6.29 | 1.90${x10}^{-11}$ | 4.86${x10}^{-8}$ |
| *Prevotella* sp. Marseille-P2931 | 0.0002 | 0.29 | -5.17 | 6.44${x10}^{-5}$ | 0.009 |
| *Prevotella stercorea* | 0.01 | 0.27 | -5.67 | 7.47${x10}^{-9}$ | 6.71x10⁻⁶ |
| *Veillonella parvula* | 0.03 | 0.15 | -2.38 | 0.0004 | 0.038 |
